# Comparison of Management for Central Venous Stenosis With or Without Previous Catheter Placement

**DOI:** 10.3389/fneur.2021.703286

**Published:** 2021-09-21

**Authors:** Wei Ma, Zhengde Zhao, Qining Fu, Liangzhu Hu, Xia Zhao, Chao Wang, Yangdong Liu

**Affiliations:** ^1^Department of Vascular Surgery, The First Affiliated Hospital of Chongqing Medical University, Chongqing, China; ^2^Department of Vascular Surgery, South China Hospital, Health Science Center, Shenzhen University, Shenzhen, China

**Keywords:** central venous stenosis, hemodialysis, central venous catheter, percutaneous endovascular intervention, patency

## Abstract

**Objective:** To compare central venous stenosis/occlusion with or without previous jugular catheter placement history.

**Methods:** Data of patients with central vein stenosis/occlusion receiving endovascular intervention in our hospital from January 2015 to December 2018 were collected and analyzed.

**Results:** Twenty-nine patients with previous jugular catheter placement history (CVC group) and 33 patients (excluded two with technical failure) without such history (non-CVC group) are included in this study. Previous jugular catheter placement history raised the risk of postintervention recurrence 1.02 times (CVC group vs. non-CVC group, HR = 2.02 95%CI: 0.91–4.48). The primary patency rate at 6, 12, 18, and 24 months was 76.9, 54.2, 45.5, and 25.0% separately in the CVC group and 80.6, 70.0, 67.9, and 44.4% separately in the non-CVC group. The assisted primary patency rate at 6, 12, 18, and 24 months was 92.3, 91.7, 86.4, and 68.8% separately in the CVC group and 93.5, 90.0, 82.1, and 61.1% separately in the non-CVC group. Patients in the CVC group received a higher frequency of reintervention (0.7 times/year/patient vs. 0.3 times/year/patient). There was no significant difference in the assisted primary patency rate between the two groups. Different primary interventions (angioplasty alone, bare metal stent, stent graft) did not affect primary patency and assisted primary patency, but percutaneous transluminal stenting (PTS) with a bare metal stent had a significant lower primary patency rate between 3 and 24 months compared with PTS with a stent graft (*p* = 0.011).

**Conclusion:** Central venous stenosis/occlusion with a previous jugular catheter placement history develops symptoms earlier and had a worse prognosis after endovascular intervention. More efforts are needed to carry out end-stage kidney disease life plan to reduce the harm of evitable catheter placement.

## Introduction

Central venous stenosis/occlusion is a knotty complication in hemodialysis patients. It could lead to venous hypertension, which may affect the quality of hemodialysis and even cause the loss of access function. Meanwhile, severe swelling may bring difficulty in access cannulation and also directly affect the quality of life (1).

Central venous hemodialysis catheter (CVC) placement is regarded as the leading cause of central venous pathology (2, 3). However, a considerable number of cases do without previous catheter placement history. For example, anatomical compression, especially left innominate vein compression, has been reported as a reason for this disease (4).

Nowadays, percutaneous intravascular intervention is recommended as the first choice for central venous stenosis/occlusion management despite the unsatisfied long-term patency (5). Considering the cases of central venous stenosis secondary to CVC placement and the other ones without previous catheter placement history might have different pathogenesis although they share some common part certainly, whether these differences play a role in the clinical manifestation and prognosis after the endovascular intervention is unknown yet. Here, we retrospectively analyzed our patients with central venous stenosis receiving endovascular intervention and divided them into two groups, one for the ipsilateral side with previous catheter placement and the others for no such history to clarify the effect of previous catheter placement history on prognosis after the endovascular intervention.

## Methods

### Study Setting and Patients

A database was maintained of all hemodialysis access patients undergoing endovascular intervention of central venous stenosis/occlusion between January 2015 and December 2018 in the Hemodialysis Access Center of Jinshan Branch, the First Affiliated Hospital of Chongqing Medical University. Excluding patients who did not have any follow-up data for all patients captured in this time period, we identified dates of access creation and previous jugular catheter placement history as well as demographics, symptoms, existing comorbid conditions, dates of percutaneous intervention, lesion location, and specific ways of intervention. Data related to all reinterventions due to recurrent central venous stenosis up to May 2019 were collected, including dates of reintervention and specific ways of management. All patients received a questionnaire on upper extremity symptoms and a physical examination for signs of stenosis/occlusion (upper limb edema, superficial vein dilation) in a clinic interview in May to July 2019 to inspect the patency of central veins (“patency” is defined below), and the symptomatic patients received further central venous venography. For patients who died before May 2019, the time and cause of death were documented. This retrospective study obtained ethical approval from the First Affiliated Hospital of Chongqing Medical University Research Ethics Review Board, and informed consent from all participants was exempted.

### Endovascular Treatment Technique

Indications for treatment were persistent moderate/severe swelling in the arm (e.g., extent of the upper limb edema whether to wrist/elbow/shoulder/entire arm with chest and face), decreasing flow during dialysis, increasing venous pressures, and change in the bruit/pulse. The single puncture technique was employed at the hemodialysis access site to enter the venous system. For a complete occlusion lesion, the femoral vein puncture approach may be also used as needed. Diagnosis was made based on contrast central venous venography before any intervention. Angioplasty balloons used in this study were INVATEC Admiral Xtreme (Medtronic, Minnesota, US) or Dorado (Bard, New Jersey, US). The selection of balloon size was determined according to the vessel diameter of the normal segments between two ends of the lesion. Intravascular stents were used in cases with elastic retraction after percutaneous transluminal angioplasty. Residual stenosis above 30% after the angioplasty was taken for indication of stent placement. Bare, self-expanding nitinol stents were deployed at early stage as LifeStent (Bard, New Jersey, US), Complete Se Vascular (Medtronic, Minnesota, US), SMART Control (Cordis, Johnson & Johnson, Florida, US). In the later period, stent grafts, Fluency Plus (Bard, New Jersey, US) were used in our center.

Access abandonment by resection or ligation of AV fistula or graft in the ipsilateral access site due to technical failure, patients' preference after recurrence, or thrombosed access without further attempts at salvage, were the end point of follow up. Data were collected on the presence of stenosis and location of the lesion, technical success rate, postintervention complications, periprocedural or 30-day death, and record of long-term patency.

### Definitions

Central venous stenosis in this study included intrathoracic venous stenosis and costoclavicular junction stenosis. Technical failure was defined as an inability to cross the lesion at the time of the primary procedure. Technical success was defined as a residual stenosis of <30% and disappearance of abnormal collateral vessels around the stenosis on venograms after the endovascular procedures. The major complication was defined as any event not routinely observed after the procedure that required a therapeutic intervention or rehospitalization within 30 days of the procedure, including arterial puncture, major bleeding, major hematoma, pneumothorax, hemothorax or mediastinal hematoma, and air embolism. Primary patency was defined as a patent central vein without recurrent stenosis or the need for further intervention within the central veins. Assisted primary patency was defined as a patent central vein that underwent further intervention to improve patency. All the definitions were in accordance with current criteria of the Society for Vascular Surgery (SVS) and the Society of Interventional Radiology (6, 7).

### Statistical Analysis

Statistical analyses were performed using SPSS software (version 25.0). Continuous values with normal distribution are reported as means ± standard deviation; continuous values with non-normal distribution are reported as median and quantile; categorical variables are reported as percentages. Postintervention hemodialysis access patency rates of the central vein are calculated using the Kaplan–Meier curve in GraphPad Prism software (version 8.0) with requirement of current SVS criteria, and long-rank tests were used to compare the difference of Kaplan–Meier curves between patients with and without previous catheter placement. Cox proportional hazard regression models were performed to investigate the association between central venous post-intervention hemodialysis access patency and history of previous catheter placement. The subgroup analysis of Kaplan–Meier curves was performed among position of lesion (left and right) and intervention approach. The comparison of central venous post-intervention patency between different intervention approaches, including percutaneous transluminal angioplasty (PTA), percutaneous transluminal stenting (PTS), bare metal stent, and stent graft (BMS), were conducted by Kaplan–Meier curve and long-rank test. Landmark analysis, which refers to the practice of designating a time point occurring during the follow-up period and analyzing only those subjects who have survived until the landmark time, were performed to evaluate the effect of morbidity at a particular landmark time point on survival up to the end of the study (8). Landmark analyses were used when two Kaplan–Meier curves met.

## Results

### Patient Population

Seventy-four patients with central venous stenosis/occlusion underwent primary percutaneous therapy during the survey interval period. Ten patients were excluded for lack of follow-up data in all study periods. Among the 64 patients with central venous stenosis/occlusion included in this study, 29 patients had a previous hemodialysis jugular catheter implantation history at the ipsilateral side of stenosis/occlusion lesion (hereinafter referred to as the CVC group), 35 patients did not have such previous hemodialysis jugular catheter implantation history at the ipsilateral side of stenosis/occlusion lesion (hereinafter referred to as the non-CVC group). Technique failure occurred in two patients of the non-CVC group, and the technique success rate was 100% and 94.3% in the CVC and non-CVC groups, respectively, without a significant statistical difference (*p* = 0.193). These two patients accepted new arteriovenous fistula in the contralateral upper limb and were not included in the following analysis. The patients in the CVC and non-CVC groups did not differ with respect to gender, age, etiology, comorbidities, and access type (Table 1). The CVC group had a significant lower proportion in the left side (34.5 vs. 81.8%, *P* < 0.001) compared with the non-CVC group. Additionally, the time interval between vascular access creation on the ipsilateral upper extremity and the central venous lesion treatment for the first time was significantly shorter in the CVC group than the non-CVC group (17.5 ± 13.7 months vs. 50.1 ± 39.7 months, *p* < 0.05).

**Table 1:** Baseline characteristics of the patients.

	**CVC group**	**Non-CVC group**	***p***
Patients Number	29	33	-
**Demographics**			
Male	48.3% (14)	51.5% (17)	0.799
Patient age (years)	63.1 ± 12.9	59.2 ± 11.7	0.215
Ipsilateral left upper extremity	34.5% (10)	81.8% (27)	<0.001
Time interval^*^ (months)	17.5 ± 13.7	50.1 ± 39.7	0.001
**Etiology**			
Primary glomerular disease	51.7% (15)	42.4% (14)	0.358
Hypertensive nephropathy	24.1% (7)	30.4% (10)	
Diabetic nephropathy	17.2% (5)	6.1% (2)	
Polycystic kidney disease	6.9% (2)	6.1% (2)	
Chronic interstitial nephritis	0	9.1% (3)	
Trauma	0	3.0% (1)	
Drug-induced kidney damage	0	3.0% (1)	
**Comorbidities**			
Hypertension	75.9% (22)	87.9% (29)	0.217
Diabetes mellitus	17.2% (5)	12.1% (4)	0.568
History of smoking	17.2% (5)	15.2% (5)	0.823
History of drinking	10.3% (3)	3.0% (1)	0.242
**AV access type**			
AVF	89.7% (26)	97.0% (32)	0.242
AVG	10.3% (3)	3.0% (1)	
**Indication for intervention**			
Swelling	93.2% (27)	97% (32)	0.577
High venous pressure	3.4% (1)	3.0% (1)	
Not specified	3.4% (1)	0	
**Position of lesion**			
Brachiocephalic vein	89.7% (26)	84.8% (28)	0.573
Subclavian vein	37.9% (11)	27.3% (9)	0.30
Superior vena cava	6.9% (2)	0	0.125
**Type of endovascular intervention**			
PTA	34.5% (10)	39.4% (13)	0.678
PTS (BMS)	34.5% (10)	39.4% (13)	
PTS (SG)	31.0% (9)	21.2% (7)	

* *Time interval: the time interval between vascular access creation on ipsilateral upper extremity and the primary treatment of central venous lesion. CVC group: patients with previous hemodialysis catheter placement in ipsilateral upper extremity; Non-CVC group: patients without previous hemodialysis catheter placement in ipsilateral upper extremity. AVF, arteriovenous fistula; AVG, arteriovenous graft; PTA, percutaneous transluminal angioplasty; PTS, percutaneous transluminal stenting; BMS, bare metal stent; SG, stent graft*.

Indications for initial intervention were not significantly different between the CVC and non-CVC groups. Considering the two groups collectively, almost all cases (59/62, 95.2%) have ipsilateral upper extremity swelling with or without associated chest, neck, and facial swelling as the clinical manifestations of central venous stenosis/occlusion; only two patients (2/62, 3.2%) showed increased venous pressures while on hemodialysis. The most common position of stenosis/occlusion lesion was the brachiocephalic vein (also known as the innominate vein) (54/62, 87%), followed by the subclavian vein (20/62, 32.2%), and the superior vena cava (2/62, 3.2%). No significant statistical differences were demonstrated between the CVC and non-CVC groups in position of central venous stenosis/occlusion lesion (Table 1). None of these patients had previously undergone any form of surgical decompression for treatment of central venous stenosis/occlusion.

There was no significant difference in the intervention approach for endovascular treatment of central venous stenosis/occlusion between the CVC and non-CVC groups (*p* > 0.05) (Table 1). On the whole, 23 patients received primary percutaneous transluminal angioplasty (PTA), and 39 patients got primary percutaneous transluminal stenting (PTS), including 23 bare metal stents (16 cases of LifeStent, 6 cases of Complete Se Vascular, and 1 case of SMART Control) and 16 stent grafts (Fluency Plus). Neither immediate nor delayed stent migration was identified in follow-up duration. There was no major complication or periprocedural or 30-day death reported in either the CVC or non-CVC group.

### Effect of Catheter Placement on Postintervention Patency

The median follow-up went for 33.5 months (23–40 months). In the CVC group, a total of 29 reinterventions were performed due to recurrence (0.7 times/year per patient) during the follow-up period, including 20 PTA and 9 PTS (4 bare stents and 5 stent grafts). In the non-CVC group, a total of 14 reinterventions were done as recurrence (0.3 times/year per patient) within the interval time of follow-up, including 11 PTA and 3 PTS (2 bare stents and 1 stent grafts).

In our study cohort, 16 patients received ligation of ipsilateral vascular access (arteriovenous fistula or arteriovenous graft) for technique failure when reintervention or patient's preference to ipsilateral vascular access abandonment and the average interval time from the initial treatment to the final ligation was 17.7 months. Five of them were in the CVC group (5/29, 17.2%), 11 cases were in the non-CVC group (11/33, 33.3%). There was no significant difference between the two groups (*p* = 0.149). By May 2019, there were 12 deaths reported. Six of them were in the CVC group, and six cases were in the non-CVC group (6/29, 20.7 vs. 6/33, 18.2%, *p* = 0.722). The reason for the 12 death cases was judged not related to central venous lesions.

**Figure 1:**
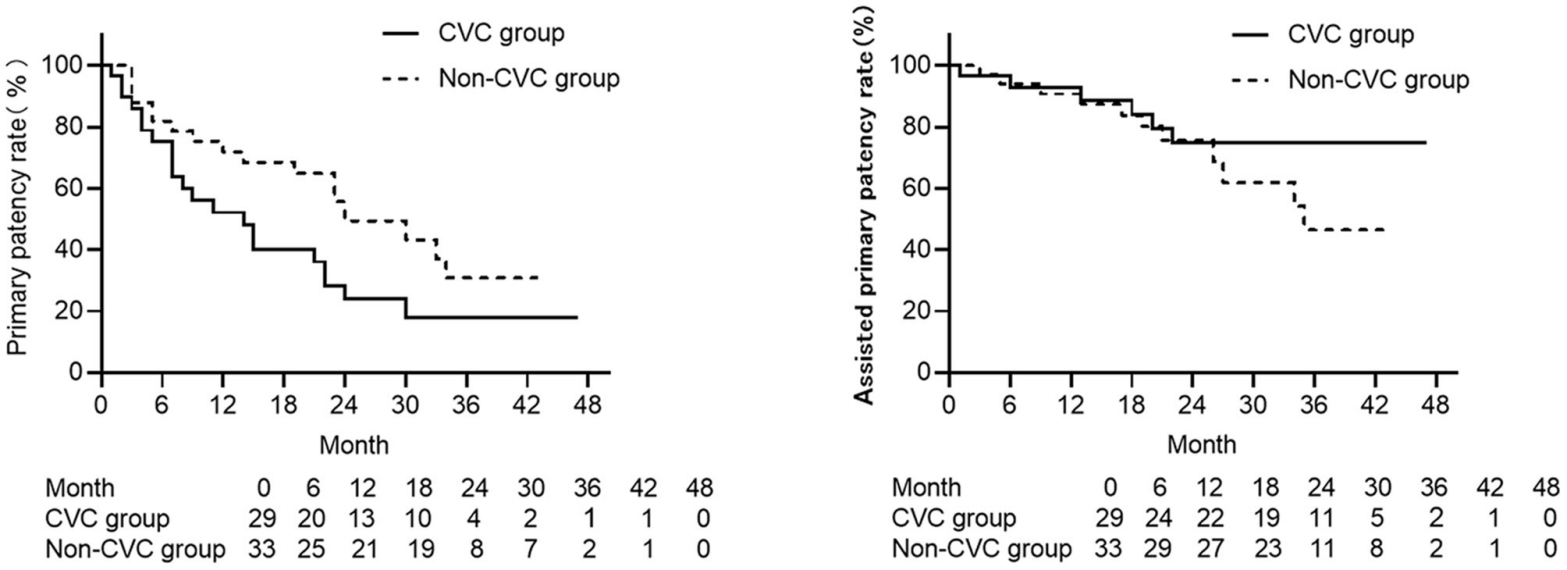
The Kaplan–Meier curves of primary patency (left) and assisted primary patency (right) for central venous lesions with (CVC group) or without (non-CVC group) previous ipsilateral central venous catheter placement history. The CVC group is depicted with solid lines and the non-CVC group with the dotted line. The CVC group has a lower primary patency compared with the non-CVC groups (*p* = 0.044), and in assisted primary patency, the non-CVC group tended to be lower after 30 months but with no significant difference (*p* = 0.725).

The primary patency rate at 6, 12, 18, and 24 months was 76.9, 54.2, 45.5, and 25.0% separately in the CVC group and 80.6, 70.0, 67.9, and 44.4% separately in the non-CVC group. Primary patency rates for the two groups are listed in Table 2. Figure 1 shows that the primary patency was lower in the CVC group compared with the non-CVC group although without a significant difference. Furthermore, when two population groups were subjected to landmark analysis for primary patency with a landmark point of 6 months, the primary patency was significantly lower in the CVC group in the period over the sixth month to the end of follow-up (*p* = 0.044) but had no statistically difference between two groups within 6 months. The assisted primary patency rate at 6, 12, 18, and 24 months was 92.3, 91.7, 86.4, and 68.8% separately in the CVC group, and 93.5, 90.0, 82.1, and 61.1% separately in the non-CVC group. Assisted primary patency rates for the two groups are listed in Table 2. No significant difference was also shown in the assisted primary patency rate between the two groups, but the assisted primary patency rate was lower in the non-CVC group after 30 months (Figure 1). A univariate Cox model showed that previous jugular catheter placement history could increase the risk of postintervention recurrence (HR=1.95, 95%CI: 1.02–3.74, *p* = 0.044). Furthermore, the result from the Cox model adjusted for the position of the lesion (left or right), and time showed previous jugular catheter placement history increased the risk of recurrence 1.02 times (CVC group vs. non-CVC group, HR = 2.02 95%CI: 0.91–4.48, *p* = 0.086), but no difference in assisted primary patency (HR = 0.71, 95%CI:0.20–2.47, *p* = 0.234) (Table 3).

**Table 2:** Primary and assisted primary patency rate between two group.

	**Primary patency rate**		**Assisted primary patency rate**	
	**CVC group**	**Non-CVC group**	**CVC group**	**Non-CVC group**
**General**				
6 months	76.9% (20/26)	80.6% (25/31)	92.3% (24/26)	93.5% (29/31)
12 months	54.2% (13/24)	70.0% (21/30)	91.7% (22/24)	90.0% (27/30)
18 months	45.5% (10/22)	67.9% (19/28)	86.4% (19/22)	82.1% (23/28)
24 months	25.0% (4/16)	44.4% (8/18)	68.8% (11/16)	61.1% (11/18)
**Subgroup: left side**				
6 months	60.0% (6/10)	76.0% (19/25)	100% (10/10)	92.0% (23/25)
12 months	50.0% (5/10)	66.7% (16/24)	100% (10/10)	87.5% (21/24)
18 months	40.0% (4/10)	65.2% (15/23)	90.0% (9/10)	82.6% (19/23)
24 months	25.0% (2/8)	46.7% (7/15)	62.5% (5/8)	66.7% (10/15)
**Subgroup: right side**				
6 months	87.5% (14/16)	100% (6/6)	87.5% (14/16)	100% (6/6)
12 months	57.1% (8/14)	83.3% (5/6)	85.7% (12/14)	100% (6/6)
18 months	50.0% (6/12)	80.0% (4/5)	83.3% (10/12)	80.0% (4/5)
24 months	25.0% (2/8)	33.3% (1/3)	75.0% (6/8)	33.3% (1/3)

*CVC group: patients with previous hemodialysis catheter placement in ipsilateral upper extremity; Non-CVC group: patients without previous hemodialysis catheter placement in ipsilateral upper extremity*.

**Table 3:** Comparison of primary patency and assisted primary patency between the patients with and without history of previous CVC placement in Cox regression model.

	**Primary patency**		**Assisted primary patency**	
	**HR (95%CI)**	***P*-value**	**HR (95%CI)**	***P*-value**
Unadjusted	1.95 (1.02–3.74)	0.044	0.56(0.20–1.63)	0.289
Adjusted^*^	2.02(0.91–4.48)	0.086	0.71(0.20–2.47)	0.588

* *Adjusted for position of lesion (right or left) and interval time*.

**Figure 2:**
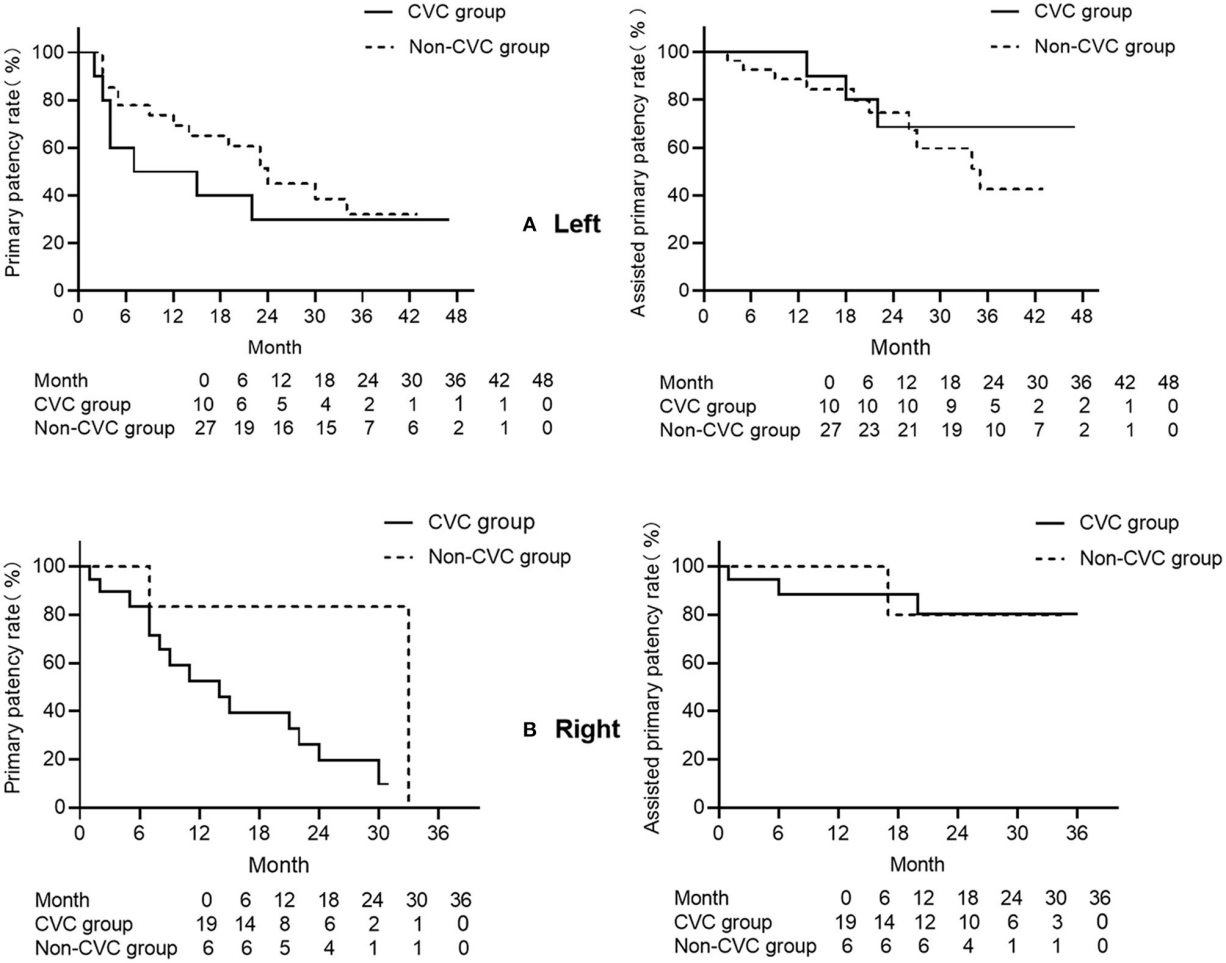
**(A)** The Kaplan–Meier curves of the primary patency (left) and assisted primary patency (right) for left-side central venous lesions with (CVC group) or without (non-CVC group) previous left central venous catheter placement history. The CVC group is depicted with solid lines and the non-CVC group with the dotted line. The primary patency in the CVC group tends to be lower after 7 months, and this tendency lasted to 30 months (*p* = 0.269). The assisted primary patency in the non-CVC group tends to be lower after 30 months (*p* = 0.725). **(B)** The Kaplan–Meier curves of the primary patency (left) and assisted primary patency (right) for right-side central venous lesions with (CVC group) or without (non-CVC group) previous right-side central venous catheter placement history. The CVC group is depicted with solid lines and the non-CVC group with the dotted line. The primary patency in the CVC group tends to be lower after 7 months (*p* = 0.900), and no significant difference is revealed between the two groups in assisted primary patency (*p* = 0.815).

The Kaplan–Meier curve was used for subgroup analysis of the patency in the left and right sides of the lesion. For the left side, the primary patency rate tended to be lower in the CVC group from the 7th to the 30th month. Nevertheless, the assisted primary patency rate in the non-CVC group was lower after 30 months (Figure 2A). For the right side, the primary patency was lower in the CVC group compared with the non-CVC group after the 7th month, and the assisted primary patency rate did not show a significant difference (Figure 2B), which might be because of the limited number of cases.

### Effect of Intervention Approaches on Postintervention Patency

**Figure 3:**
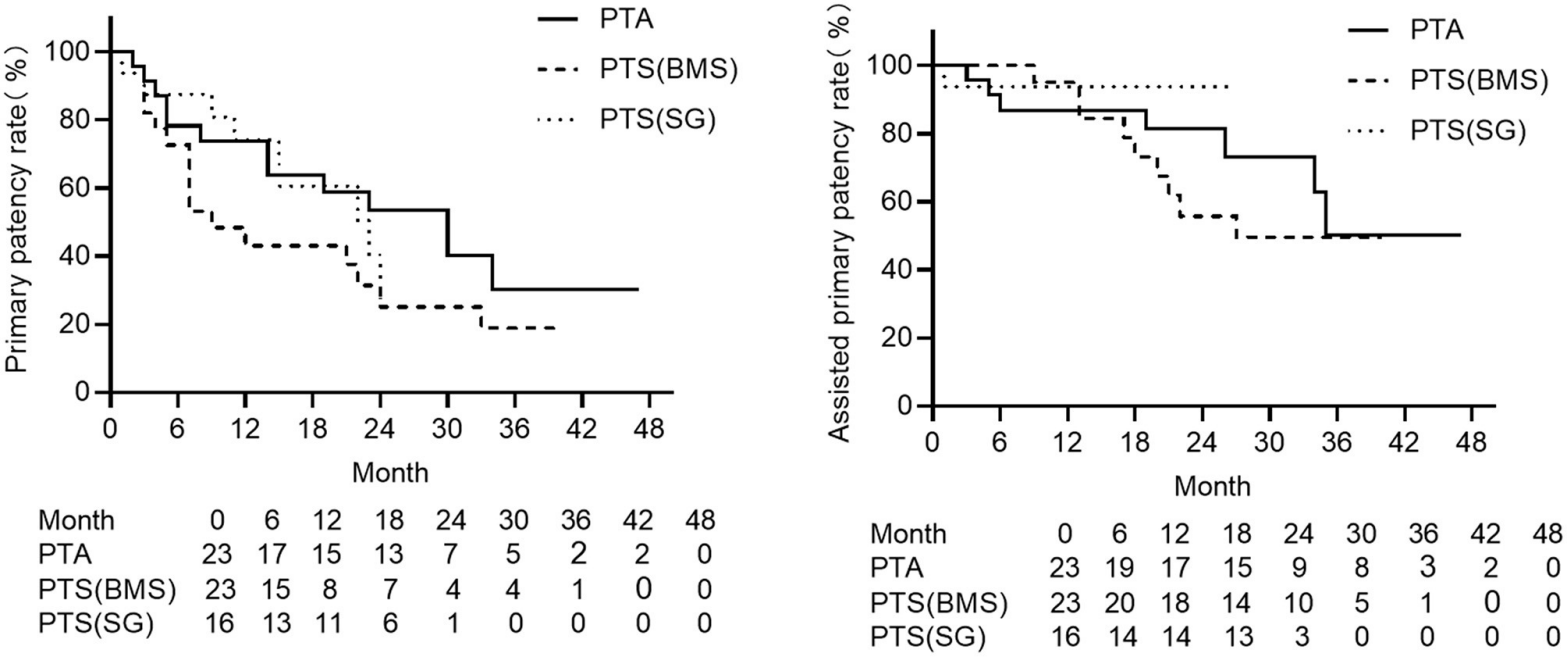
The Kaplan–Meier curves of the primary patency (left) and assisted primary patency (right) for different interventions for central venous lesions. PTA is depicted with solid lines, PTS with bare stent with short segments, and PTS with stent graft with the dotted line. PTS with bare metal stent had a lower primary patency among 3 to 24 months compared with PTS with stent graft (*p* = 0.011), and a lower primary patency after 13 months compared with PTS with stent graft (landmark analysis, *p* = 0.033).

For different primary interventions, patency rates are also shown in Table 4. No matter whether primary or assisted primary patency, PTA was neither significantly different with PTS with a bare-metal stent (*p* = 0.139 for primary patency, 0.520 for assisted primary patency), nor with PTS with a stent-graft (*p* = 0.843 for primary patency, 0.316 for assisted primary patency). Landmark analysis showed PTS with a bare-metal stent had a lower primary patency rate between 3 and 24 months (*p* = 0.011) (Figure 3, Supplementary Figure 2A) and a lower assisted primary patency rate after 13 months (*p* = 0.033) compared with PTS with a stent-graft (Figure 3, Supplementary Figure 2B).

**Table 4:** Primary and assisted primary patency rate for different primary interventions.

**Patency rate**		**6 months**	**12 months**	**18 months**	**24 months**
Primary	PTA	77.3% (17/22)	75.0% (15/20)	72.2% (13/18)	58.3% (7/12)
	PTS (BMS)	75.0% (15/20)	42.1% (8/19)	38.9% (7/18)	27.8% (4/18)
	PTS (SG)	86.7% (13/15)	73.3% (11/15)	64.3% (9/14)	25.0% (1/4)
Assisted primary	PTA	86.4% (19/22)	85.0% (17/20)	83.3% (15/18)	75.0% (9/12)
	PTS (BMS)	100% (20/20)	94.7% (18/19)	77.8% (14/18)	55.6% (10/18)
	PTS (SG)	93.3% (14/15)	93.3% (14/15)	92.9% (13/14)	75.0% (3/4)

*PTA, percutaneous transluminal angioplasty; PTS, percutaneous transluminal stenting; BMS, bare metal stent; SG, stent graft*.

Subgroup analysis was done for different primary interventions using Kaplan–Meier analysis and landmark analysis. For PTA, landmark analysis showed lower primary patency in the CVC group between 3 and 29 months (*p* = 0.041), and the assisted primary patency rate tended to drop after 24 months but without any statistical difference (*p* = 0.222) (Supplementary Figures 1A and 2C). For PTS with a bare metal stent, the primary patency rate dropped faster after 3 months (landmark analysis, *p* = 0.039) in the CVC group, and no such tendency could be noticed in the assisted primary patency rate (Supplementary Figures 1B and 2D). For PTS with a stent graft, the primary patency rate also dropped lower after 9 months in the CVC group but was not significant, and assisted the primary patency rate did not reveal a significant difference (Supplementary Figure 1C).

## Discussion

Central venous stenosis/occlusion is not a rare complication for hemodialysis patients. The incidence has been reported as 9 to 51% (9).

The pathogenesis of central venous stenosis has been attributed to two major factors: one is existing underlying stenosis, which might be caused by previous central venous catheterization, (2, 3) anatomical compression, (4) or other causes. The other is the high-flow state after the creation of arteriovenous access with resultant regions of increased turbulence (10). In our study, more right-side lesions occurred in the CVC group in keeping with more catheters placed in the right jugular vein in clinical practice although more left-side lesions occurred in the non-CVC group, which is accordant with research about left innominate vein compression by sternum and aorta ascendence, aortic arch, or brachiocephalic trunk.

Our study indicates that patients in the CVC group had a shorter time interval between vascular access creation on the ipsilateral upper extremity and the central venous lesion treatment for the first time, and this reconfirms that the central venous lesion related to the hemodialysis catheter has already developed there when the catheter is reserved. It is widely accepted that CVC placement is one of the most important risk factors for central venous stenosis/occlusion (3, 11). CVC placement could lead to vascular injury and inflammation, (2, 12) meanwhile bringing turbulent flow (13). Several factors take part in the pathogenesis, including the position of the foreign body against the vessel walls, activation of the coagulation system, the uremic milieu, and consequent inflammation. All these further result in neointimal hyperplasia (14, 15) and finally give rise to central venous stenosis/occlusion.

Our study also shows that the CVC group had a poorer long-term primary patency after 6 months, and this is not related to the side of the lesion and the time interval of symptom onset. All this highlights that CVC placement is not only a risk factor for central venous problems, once it develops symptoms, the prognosis is also worse. Patients are more likely to have recurrence and have to receive more frequent reinterventions than patients without CVC placement history. Compared with the pathogenesis of central venous disease secondary to the catheter, studies on non-CVC-related central venous disease are limited, and molecular and cellular level study is needed to understand the differences between them.

The assisted primary patency of PTA, PTS (BMS), and PTS (SG) at 6, 12, 18, and 24 months are 86.4, 85.0, 83.3, and 75.0%; 100, 94.7, 77.8, and 55.6%; and 93.3, 93.3, 92.9, and 75.0%, respectively. The results reveal that endovascular management could reach an acceptable assisted primary patency. There was no major complication or periprocedural or 30-day death reported in either the patients receiving PTA or patients receiving PTS. Thus, endovascular management is a safe and effective therapy for central venous disease compared with open surgery.

Regarding different primary interventions, our study shows no difference in primary patency rates between PTA and PTS groups, which is in accord with studies by Bakken (16) and Wu (17). A similar suggestion could be drawn as Ozyer's study gives (18) if, without obvious residual stenosis, stent deployment could not improve patency after angioplasty.

However, PTS with stent-graft got an improved primary patency rate at least between 3 and 24 months compared with PTS with a bare-metal stent in our study. That a stent graft brings better long-term patency has been confirmed by Quaretti et al. (19). In their study, the primary patency rate at 24 months of stent-graft was 84%, and that of a bare metal stent was only 46%. As the restriction of available equipment, we used a bare-metal stent at the early stage of our study and then switched to a stent-graft. The follow-up for a stent-graft is shorter than a bare-metal stent, this limitation makes us unable to clarify long-term changes. The stent-grafts used in our study were Fluency. The study reminds us that another stent-graft, Viabahn (W. L. Gore & Associates, Delaware, US), with better flexibility compared with Fluency, better fits the original anatomical structure of vessels and might elevate the patency rate further (20).

Subgroup analysis was done in our study, and some tendencies were noticed based on Kaplan–Meier analysis, but because of the limited number of cases, especially for patency rates after 24 months, the conclusion could not be given at the moment. More studies are still needed.

Finally, our study provides a new perspective about the harm of a hemodialysis catheter. The Kidney Disease Outcomes Quality Initiative (KDOQI) Clinical Practice Guideline for Vascular Access: 2019 update introduces a new concept as the end-stage kidney disease life plan emphasizes that each patient with progressive CKD and/or with an eGFR 15–20 mL/min/1.73 m^2^ or already on kidney replacement therapy should have an individualized ESKD life plan that is regularly reviewed, updated, and documented on their medical record (21). Although the 2019 update no longer uses “fistula first, catheter last,” as it does not suit everyone, the new concept life plan, in fact, states for patients who have a better choice than catheter, a plan is needed to guarantee patients are free from the harm of evitable catheter placement. Since the “fistula first” initiative, the situation has been greatly improved for catheter abuse. An end-stage kidney disease life plan creates a higher criterion for us.

This study has several limitations. Because the patient sample was small, and follow-up time was short, we could not get statistically significant differences for some points. Particularly, small numbers of patients in each intervention group (balloon, stent, stent-graft) contribute a likelihood of a type II statistical error when calculating comparisons. Though further study with large samples is needed to overcome the defect of sample size, this study still provides the new insights of the difference of long-term patency on central venous stenosis/occlusion lesions with or without a previous jugular catheter placement history. Besides this, the follow-up duration is relatively short, and this might result in the conclusion in our study not being suitable for the patient with the postintervention exceeding 24 months. As far as we know, primary patency and assisted primary patency of central venous stenosis/occlusion after endovascular treatment above 24 months should not exceed 20 and 35%, (17) so the comparison of patency between groups within 24 months could provide us the knowledge of the effect of previous jugular catheter placement history on postintervention patency under circumstances lacking sufficient evidence at present. As a single-center retrospective study, interventions were employed mainly based on experiences, devices available at that time, and the surgeon's personal preference. Meanwhile other factors, such as financial situation or personal prejudice affected patients' choice of access ligation. All these might influence our results. More multicenter research with a large sample size, long follow-up duration, and several added variables, such as financial situation and personal prejudice are necessary to carry out to sufficiently comprehend the role of previous jugular catheter placement history on postintervention patency.

## Conclusion

Central venous stenosis/occlusion with previous jugular catheter placement history is more likely to locate in the right side and develop symptoms earlier. Endovascular intervention is safe and effective, but compared with lesions without previous catheter placement history, lesions with such history have a poorer long-term primary patency and receive a higher frequency of reintervention. More efforts are needed to carry out the end-stage kidney disease life plan to reduce the harm of evitable catheter placement.

## Data Availability Statement

The raw data supporting the conclusions of this article will be made available by the authors, without undue reservation.

## Ethics Statement

The studies involving human participants were reviewed and approved by First Affiliated Hospital of Chongqing Medical University Research Ethics Review Board. Written informed consent for participation was not required for this study in accordance with the national legislation and the institutional requirements.

## Author Contributions

The project was mainly performed by WM and QF who drafted the original manuscript. Statistical support and critically review of the paper was provided by QF and ZZ. ZZ provided great support in manuscript revision. The project was partly performed by LH, XZ, and CW. This project led by YL who contributed to the design of this project and critical review of manuscript. All authors contributed to the article and approved the submitted version.

## Funding

The study was supported by Health and Family Planning Commission of Chongqing (2016ZDXM004).

## Conflict of Interest

The authors declare that the research was conducted in the absence of any commercial or financial relationships that could be construed as a potential conflict of interest.

## Publisher's Note

All claims expressed in this article are solely those of the authors and do not necessarily represent those of their affiliated organizations, or those of the publisher, the editors and the reviewers. Any product that may be evaluated in this article, or claim that may be made by its manufacturer, is not guaranteed or endorsed by the publisher.
