# Supplementary material for: Comparison of Management for Central Venous Stenosis With or Without Previous Catheter Placement
Source: Front Neurol. 2021 Sep 21;12:703286. doi: 10.3389/fneur.2021.703286 (PMC8490807; doi:10.3389/fneur.2021.703286)

Supplemental Figure 1

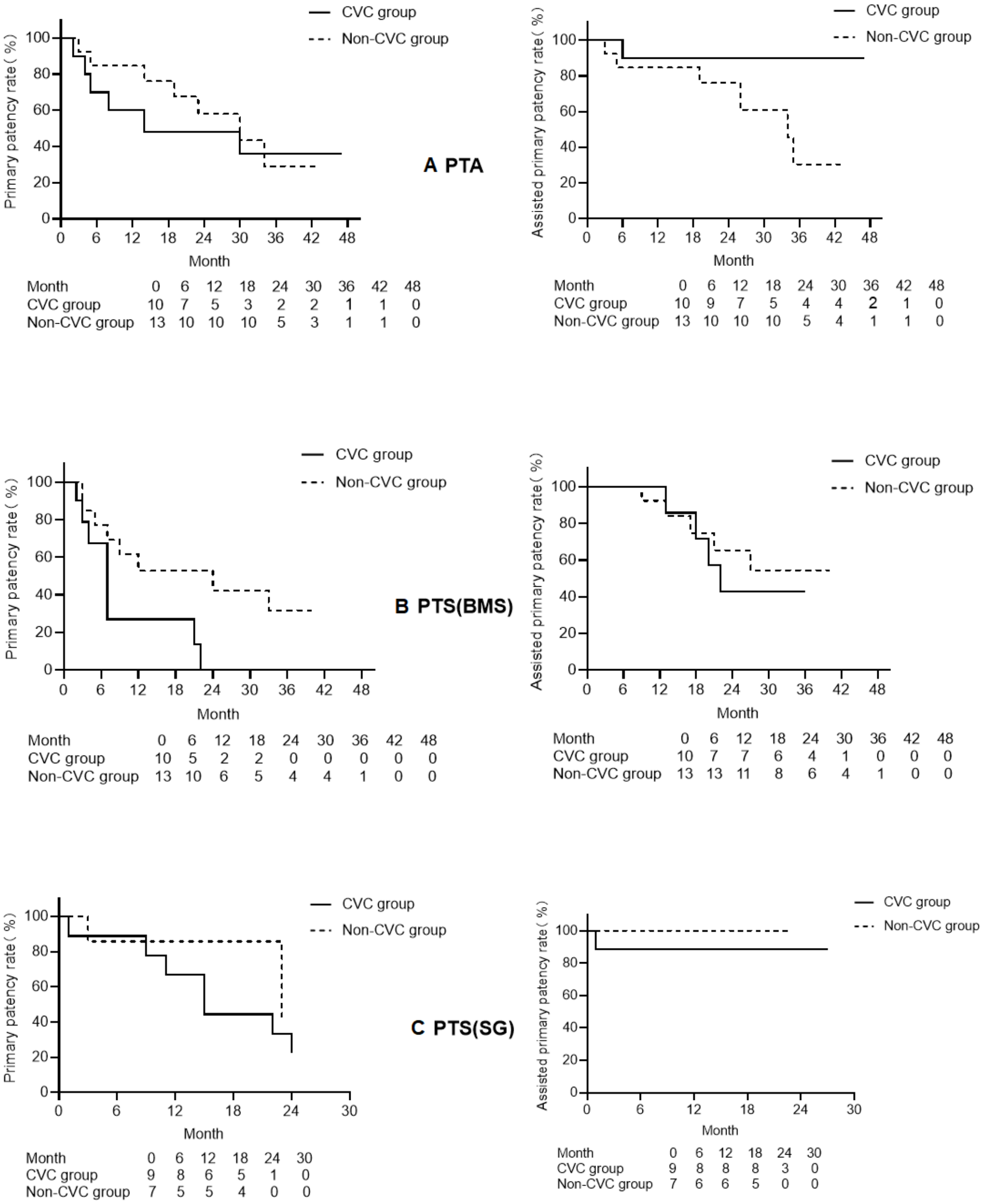

Supplemental Figure 2

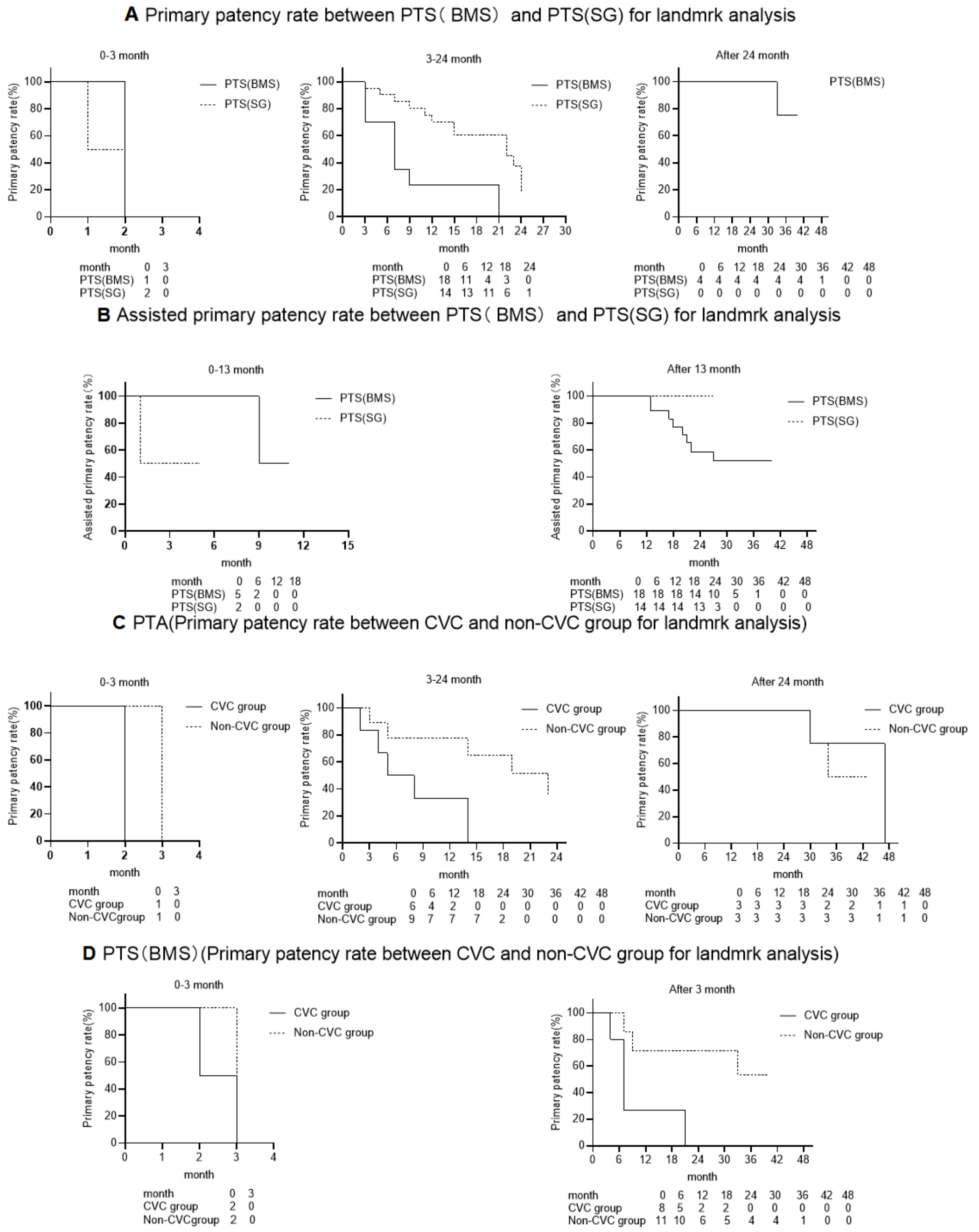

Supplement: Supplementary file 1 [file Image_1.pdf]
